# Pan-Cellulosomics of Mesophilic Clostridia: Variations on a Theme

**DOI:** 10.3390/microorganisms5040074

**Published:** 2017-11-18

**Authors:** Bareket Dassa, Ilya Borovok, Vincent Lombard, Bernard Henrissat, Raphael Lamed, Edward A. Bayer, Sarah Moraïs

**Affiliations:** 1 Department of Biomolecular Sciences, The Weizmann Institute of Science, Rehovot 7610001, Israel; bareket.dassa@weizmann.ac.il; 2 Department of Molecular Microbiology and Biotechnology, Tel Aviv University, Ramat Aviv 6997801, Israel; ilyabo@tauex.tau.ac.il (I.B.); lamedr@post.tau.ac.il (R.L.); 3 Architecture et Fonction des Macromolecules Biologiques, CNRS and Universite Aix-Marseilles I & II, Marseilles 13288, France; vincent.lombard@afmb.univ-mrs.fr (V.L.); bernard.henrissat@afmb.univ-mrs.fr (B.H.); 4 Faculty of Natural Sciences, Ben-Gurion University of the Negev, Beer-Sheva 8499000, Israel

**Keywords:** cellulosomes, cohesin, dockerin, scaffoldin, glycoside hydrolases

## Abstract

The bacterial cellulosome is an extracellular, multi-enzyme machinery, which efficiently depolymerizes plant biomass by degrading plant cell wall polysaccharides. Several cellulolytic bacteria have evolved various elaborate modular architectures of active cellulosomes. We present here a genome-wide analysis of a dozen mesophilic clostridia species, including both well-studied and yet-undescribed cellulosome-producing bacteria. We first report here, the presence of cellulosomal elements, thus expanding our knowledge regarding the prevalence of the cellulosomal paradigm in nature. We explored the genomic organization of key cellulosome components by comparing the cellulosomal gene clusters in each bacterial species, and the conserved sequence features of the specific cellulosomal modules (cohesins and dockerins), on the background of their phylogenetic relationship. Additionally, we performed comparative analyses of the species-specific repertoire of carbohydrate-degrading enzymes for each of the clostridial species, and classified each cellulosomal enzyme into a specific CAZy family, thus indicating their putative enzymatic activity (e.g., cellulases, hemicellulases, and pectinases). Our work provides, for this large group of bacteria, a broad overview of the blueprints of their multi-component cellulosomal complexes. The high similarity of their scaffoldin clusters and dockerin-based recognition residues suggests a common ancestor, and/or extensive horizontal gene transfer, and potential cross-species recognition. In addition, the sporadic spatial organization of the numerous dockerin-containing genes in several of the genomes, suggests the importance of the cellulosome paradigm in the given bacterial species. The information gained in this work may be utilized directly or developed further by genetically engineering and optimizing designer cellulosome systems for enhanced biotechnological biomass deconstruction and biofuel production.

## 1. Introduction

The plant cell wall forms a complex structure of cellulose fibers embedded into a colloidal mixture of hemicellulose, pectin, and lignin [1]. Cellulolytic microorganisms are prevalent in natural lignocellulose-containing habitats abundant in plant cell walls, such as soil, wood, rumen, and termite guts, or in man-made sewage sludge or compost piles [2,3,4]. They employ various strategies to efficiently hydrolyze cellulose and hemicellulose of wood and plants into simple hexose and pentose sugars that will be directed to their carbohydrate metabolism and cell construction [5]. One strategy for fiber deconstruction selected by various aerobic or anaerobic bacteria and fungi, is the secretion of multiple degradative enzymes in the free state (such as cellulases, hemicellulases, and ligninases) [6]. Remarkably, some anaerobic bacteria evolved a different strategy for an efficient degradation of plant cell wall polysaccharides, which is the production of multiple interactive enzymes and structural proteins that assemble together in large enzymatic complexes, termed *cellulosomes* [2,7].

Cellulosomes are extracellular structures that generally display the carbohydrate-active enzymes on the bacterial cell surface via their attachment in a well-organized complex. Cellulosomes are constructed from two major types of components: a non-enzymatic *scaffoldin* structural protein which contains multiple copies of *cohesin* modules, and multiple *dockerin*-containing degradative enzymes, that are incorporated into the scaffoldin via a strong and specific inter-modular cohesin–dockerin interaction (K_a_ > 10^11^ M^−1^). Thus, in the cellulosome, multiple and heterogeneous enzyme types (such as endoglucanases, cellobiohydrolases, and xylanases) can act synergistically in close proximity. In addition, the major scaffoldin generally mediates the attachment of the complex to both the cellulosic substrate, via a carbohydrate-binding module (CBM), and the cell surface, via divergent type of cohesin–dockerin integration into an anchoring protein.

Cellulosome architectures vary greatly among the cellulosome-producing bacterial species, but two global types of architectures of cellulosome systems have been observed, namely simple and complex [2,7]. Simple cellulosomes have so far been observed in mesophilic clostridial species, such as *Clostridium cellulovorans*, *C. cellulolyticum*, *C. josui,* and in the apparently inactive cellulosome of *C. acetobutylicum* [8,9,10,11]. The *simple* cellulosome architecture, includes a *single* scaffoldin protein, harboring an N-terminal family 3 CBM (CBM3) [12], and is composed of repeating cohesins and X2 modules [13]. The cohesins of the major scaffoldin integrate various dockerin-bearing enzymes, some of which are coded on the genome in an *enzyme-gene cluster*, downstream of the major scaffoldin gene. On the other hand, *complex* cellulosome systems contain *multiple* scaffoldin proteins, having a CBM3, which is located internally on the major scaffoldin. The intermodular linkers in the major scaffoldins are relatively long, Pro/Thr-rich sequences [14,15]. The major scaffoldin genes are clustered in the genome in a *sca gene cluster*, wherein the sca genes do not appear adjacent to the dockerin-bearing enzymes, as they do in the simple cellulosome-producing bacteria [16]. Complex cellulosomes have been observed in *C. thermocellum* [17], *Bacteroides* (*Pseudobacteroides*) *cellulosolvens* [18,19], *Acetivibrio cellulolyticus* [20,21], *Ruminococcus flavefaciens* [22,23], and *C. clariflavum* [24,25].

In 2004, the foundation of a genomic comparison of cellulosomal genes was established, based on the hypothesis that cellulosome-producing bacteria in anaerobic habitats are under selective pressure to evolve a superior type of efficient strategy for cellulose degradation [7]. Since 2007, whole-genome sequences of mesophillic cellulosome-producing *Clostridium* species gradually became available for comparative analyses [6,26,27], with great interest in their *cip*-*cel* operon, their enzymatic collection, and the putative regulation of their respective cellulosome components [28]. Recent genome sequencing efforts allowed us to perform a detailed analysis of the cellulosomal blueprint of several cellulosome-producing bacteria, therefore expanding considerably the original observations and enabling a broader perspective of the field of “cellulosomics”. We also report here, for the first time, the presence of multiple cellulosomal elements in *C. sufflavum*, *C. termitidis*, and *C. saccharoperbutylacetonicum*, thus both defining them as potential cellulosome producers and expanding the prevalence of the cellulosomal paradigm in nature.

In the present work, we systematically identified and compared hundreds of cellulosomal genes (scaffoldins, enzymes, and regulatory elements), which evolved in a dozen mesophillic cellulosome-producing clostridia. High conservation in global genomic features was observed among the species, such as the organization of the cellulosome gene cluster, and the basic sequence properties of cohesins/dockerins, including similarities in recognitions residues. Yet, variations in the function, number and organization of cellulosomal elements suggest the evolution of a species-specific cellulosome blueprint.

## 2. Materials and Methods

### 2.1. Genomes Sequences

Draft genomes of eleven cellulose-degrading and mesophilic bacteria were analyzed in this study. The GenBank accession numbers of the draft genomes are detailed in Table 1.

### 2.2. Bioinformatic Identification of Cellulosomal Components

Prediction of cohesins and dockerin sequences in draft genome assemblies of *C. papyrosolvens* DSM 2782 and *C. papyrosolvens* C7 was done using BLAST [29], with known cohesin and dockerin sequences as queries (i.e., those of *C. thermocellum*, *C. cellulovorans*, or *Acetivibrio cellulolyticus*). Hits of E-value < 10^−4^ were individually examined. Carbohydrate-active enzymes were identified using CAZy [30], a comprehensive resource for carbohydrate-active enzymes which uses BLAST, or using hidden Markov models (HMMs) to classify proteins to families of glycoside hydrolases, carbohydrate esterases, polysaccharide lyases, carbohydrate-binding modules, and glycosyl transferases. Additional functional modules were identified using CD-search [31]. Multiple sequence alignments of cohesins and dockerins were generated using ClustalO [32]. Weblogos of dockerin sequences were constructed using WebLogo 2.8.2 [33]. The accession numbers or ORFs of the identified dockerin-containing proteins are listed in Supplementary Table S1, and identification of their signal peptide was done using SignalP 4.1 server (http://www.cbs.dtu.dk/services/SignalP-4.1/ for Gram-positive bacteria). Whole-genome comparison was done using the SEED viewer at RAST server [34]. Cohesin dendrograms were constructed using PhyML 3.0 [35], where branches below 80% bootstrapping were collapsed.

## 3. Results

### 3.1. Comparative Characteristics of the Mesophilic Clostridia Cellulosomal Systems 

In this work, we compared and analyzed a group of related mesophiles that genetically encode for dozens of cellulosomal elements, inhabiting diverse niches, such as soil, wood, and rumen (Table 1). These include previously reported species, such as *C. acetobutylicum* [36], *C. josui* [37], *C. papyrosolvens* [38], *C. cellulolyticum* [39], *C. cellulovorans* [40], *C. cellobioparum* [41], and *Clostridium* sp. strain BNL1100 [42]. In addition, we analyzed newly sequenced organisms, such as *C. bornimense* M2/40 [43,44], *C. saccharoperbutylacetonicum* [45], *C. termitidis* (from termites *Nasutitermes lujae*) [46], and *C. sufflavum* [47], in which we first report, here, the presence of cellulosomal elements.

For profiling the cellulosomal system of each genome, we focused on its specific properties: the *number* of scaffoldins and dockerin-containing proteins which are potentially coded; the *nature* of the cellulosomal protein modules (i.e., types of cohesins, dockerins, and breakdown of CAZymes into categories); and genomic *organization* and sequence conservation of genes coding for cellulosomal components.

The cellulosomal systems that were observed reflect different degrees of complexity (Table 1). Small variations were observed in the number of cohesins, ranging from 3 cohesins in *C. saccharoperbutylacetonicum* up to 15 in *C. sufflavum*, with the number of scaffoldins varying from 2 to 7.

The majority of the examined species code for only 2–3 scaffoldins, while *C. cellulovorans*, *C. termitidis* and *C. sufflavum* code for more than 5 scaffoldins (although some of which may result from incorrect or inadequate assembly of the genome). However, great variation was observed in the number of dockerin-bearing proteins, whereby *C. saccharoperbutylacetonicum*, *C. bornimense*, and *C. acetobutylicum* code for strikingly few dockerins (≤10), whereas other species contain a range of 28–88 dockerin-containing proteins. Similarly, 2–3-fold variation was also observed in the total number of CAZymes coded in the genome, ranging from 60 enzymes (*C. bornimense*) to 218 (*C. termitidis*). Nevertheless, when considering draft genomes, the number of scaffoldins may have been underestimated; dockerin-containing protein numbers would also be affected to a lesser extent. Moreover, assembly issues (especially in draft genomes) may result in gene duplication and distortion in numbers and disposition of repeated modular components, such as cohesin and X2 modules.

### 3.2. Conserved Patterns in the Orthologous Sca Gene Cluster 

**Figure 1:**
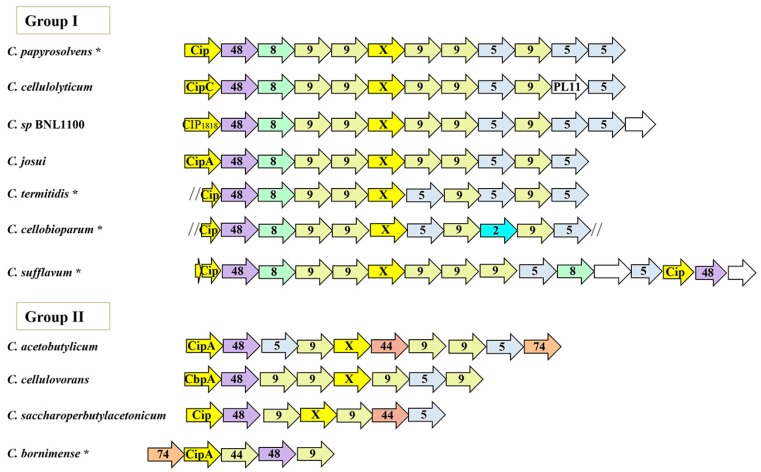
Similar and modular organization of the cellulosomal gene clusters (*sca*) of mesophiles. Schematic representation of the gene cluster harboring the major scaffoldin, and followed by genes coding for dockerin-containing cellulolytic enzyme, which are organized in a similar sequence along the gene cluster of the marked species. The major scaffoldin gene is represented by *cip*; numbers denote the family of glycoside hydrolases; X stands for the *orfX* gene; asterisks (*) mark draft genomes that have more than two contigs; slashes (//) indicate that the ORF may not be complete, because it was located at the end of contig.

In all the examined species, the major scaffoldin gene, termed *cip* (originally referred to as “cellulosome-integrating protein”), is typically organized on the chromosome in a large cluster of 5 to 16 genes, with most species having 10 to 12 genes (Figure 1), in which the *cip* gene is the first gene. It is followed downstream by genes coding for cellulolytic enzymes, belonging to GH families 48, 9, and 5, which play key roles in cellulose cellulosomal degradation [48,49,50]. In between the genes of the cluster lies a conserved gene, termed *orfX*, which codes for a cohesin-containing protein (up to 97% sequence similarity among the mesophilic bacterial species). The overall gene organization of the cluster is comparable in all species, suggesting that the cellulosomes of the mesophilic bacteria originated from a common ancestor. Nevertheless, we still observed two patterns of gene architectures among the different bacteria. We divided the species in two groups based on this gene cluster organization (Figure 1). The Group I mesophilic clostridia have an identical organization of their six first genes, which encode for the major scaffoldin (Cip), followed by the GH8 enzyme, two GH9s, and the mysterious cohesin-containing OrfX protein. Thereafter, minor swapping of GH5 and GH9 enzymes ensue. An additional gene could be found in unique species, such as the *C. cellulolyticum* gene cluster that contains a singular PL11 gene at the 3′-end of the cluster. The cluster organization is more conserved in the genomes of closely related cellulolytic bacteria, such as *C. cellulolyticum*, *Clostridium* sp. BNL1100 and *C. josui*. Intriguingly, *C. sufflavum* presents two copies of the *cip* and the GH48 genes, which may be the result of a gene duplication event. In contrast, group II species do not contain a GH8 gene, and instead display a GH74 or GH44 gene. Remarkably, *C. bornimense* is the only species coding for an enzyme at the 5′-end of the cluster, upstream to the *cip* gene [51].

### 3.3. Modular Organization of the Major Scaffoldin Gene

**Figure 2:**
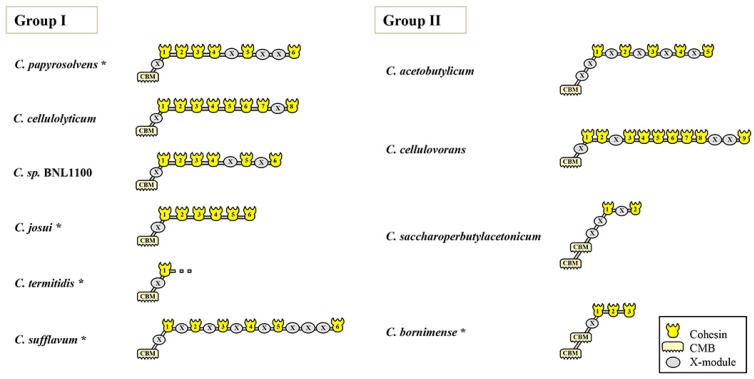
Modular and domain architectures of the primary scaffoldins of mesophilic cellulosome-producing bacteria. Schematic representation of the functional protein modules comprising the primary scaffoldin protein of cellulosome-producing mesophiles. Slashes (//) denote the end of a contig. Asterisks (*) mark draft genomes that have more than two contigs. GenBank accession numbers for the scaffoldins are as follows: *C. papyrosolvens*, 325985039; *C. cellulolyticum*, AAC28899.2; *Clostridium* sp. BNL1100, 373945107; *C. josui*, 640241850; *C. cellulovorans*, 302578508; *C. acetobutylicum*, 336290364; *C. acetobutylicum*, 15894197; *C. acetobutylicum*, 325508325; *C. saccharoperbutylacetonicum*, 451784659; *C. termitidis*, 474480363; *C. sufflavum*, Ga0056032 and *C. bornimense*, 584458187. *C. cellobioparum* was omitted, because the gene encoding its scaffoldin was fragmented in the draft genome sequence.

The modular organization of the mesophilic clostridia shows both striking similarity and intriguing variety among the species. Evaluation of the relationship between cohesins can be exemplified for *C. papyrosolvens*, in which sequence analysis identified a 137 kDa scaffoldin protein, bearing an N-terminal CBM3 followed by six type-I cohesin modules, which are interspersed with conserved X2 modules (Figure 2). Most of the scaffoldins from the other clostridial species contain a CBM3 at the N-terminus, six scaffoldins, and exhibit modular protein architectures strikingly similar to that of *C. papyrosolvens*, with permutations in the number and position of the X2 modules. A similar architecture is also conserved in the CipC protein of *C. cellulolyticum* and CbpA of *C. cellulovorans*, but the latter scaffoldins contain eight and nine cohesins, respectively. In *C. saccharoperbutylacetonicum* and *C. bornimense*, the scaffoldins contain two and three cohesins respectively. The number of scaffoldin-borne X modules range from one in *C. josui* to eight in *C. sufflavum*. Most of the scaffoldins exhibit a trimodular CBM3-X2-Coh at their N-terminus, except *C. acetobutylicum* and *C. saccharoperbutylacetonicum*, that bear two X2 domains between their CBM3 and Coh modules. Intriguingly, the scaffoldins of *C. saccharoperbutylacetonicum* and *C. bornimense* exhibit two copies of the CBM3 at the N-terminus. The *cip* gene is incomplete in the draft sequences of *C. cellobioparum* and *C. termitidis*, where their sequences are either interrupted or truncated at the end of the contig of the draft genome.

### 3.4. Regulation of the Sca Gene Cluster by a Conserved σ^A^-Dependent Promoter

**Figure 3:**
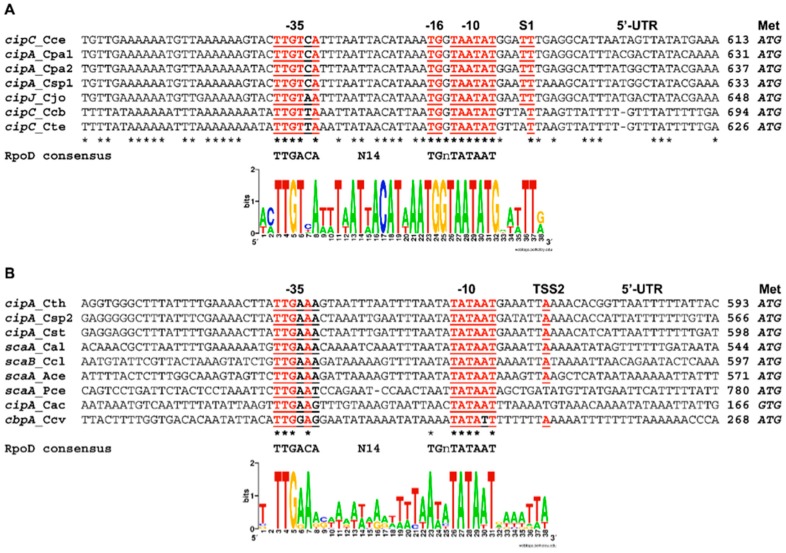
Sequence conservation of the major σ^A^-dependent promoters upstream of the respective *cip* gene cluster. (**A**) The σ^A^ (RpoD)-dependent promoter and cognate transcription start site (S1) have been experimentally identified as a major region of the *C. cellulolyticum* H10 *cipC* gene [52] and its orthologs [54]. The two T nucleotides of S1 are underlined, as well as sequences predicted to be −35, −16 and −10 elements of the *cipC* promoter; (**B**) aligned sequences are related to the recently identified RpoD-dependent promoter of the *C. thermocellum cipA* gene [54]. TSS2 is a transcriptional start site position, while −35 and −10 elements are elements of the *cipA* promoter. In both panels (**A** and **B**), 5′ UTR (untranslated regions) are shown partially, and numbers between the last nucleotide of each sequence and the predicted initial codon for methionine (Met) are provided. The two WebLogos were generated, with the sequences shown in each alignment, and they suggest putative promoter consensuses in the two groups of cellulolytic species. The promoter has two patterns of conservation, one in the related mesophiles, and a second in thermophiles and other complex cellulosomes (denoted ^†^ in designated species as follows). Cce, *C. cellulolyticum*; Cpa1, *C. papyrosolvens* DSM 2782; Cpa2, *C. papyrosolvens* C7; Csp, *Clostridium* sp. strain BNL1100; Cjo, *C. josui*; Ccb, *C. cellobioparum*; Cte, *C. termitidis*; Cth ^†^, *C. thermocellum* DSM 1313; Cst ^†^, *C. straminisolvens* JCM 21531; Ccl, *C. clariflavum* DSM 19,732; Ace ^†^, *Acetivibrio cellulolyticus* CD2; Ccv, *C. cellulovorans*; Cac, *C. acetobutylicum*; Pce ^†^, *Pseudobacteroides* (*Bacteroides*) *cellulosolvens* ATCC 35603 (DSM 2933). Asterisks (*) indicate sequenced positions with identical nucleotides.

Remarkably, the 5′-upstream region of the first *cip* gene in each cluster is conserved among all the species. This region was previously reported as the *cip–cel* operon promoter, which undergoes transcriptional regulation [52]. This conserved putative promoter sequence, upstream of the major scaffoldin gene, ranges from 862 bp in *C. termitidis* to 1286 bp in *C. cellobioparum* (Figure 3). Previously, Abdou and colleagues [52] reported an unusually remote promoter of the *cipC* gene in *C. cellulolyticum* ATCC 35319 (ortholog of the H10 strain). In that study, a single σ^A^-dependent promoter (P1) was determined between nucleotides -671 and -643 with respect to the ATG start codon, generating a 638 nt 5′-UTR (untranslated region) of the *cipC* mRNA. A recent mRNA-seq study suggests that the *C. cellulolyticum sca* gene cluster functions as an operon, and confirms that a single promoter is located at the 5′-end of cipC [28]. The primary *cip–cel* transcript harbors at least five post-transcriptional processed sites, and suggests a post-transcriptional regulatory model for cellulosomal loci.

We used the *C. cellulolyticum cipC* 5′UTR sequence as a query to mine available genomes of mesophilic cellulosome-producing bacteria, and found an extraordinary conservation of a putative promoter motif very far from the predicted start codon of the major scaffoldin gene in the following species: *C. josui*, *C. papyrosolvens*, *C. cellobioparum*, *Clostridium* sp. strain BNL1100, and *C. termitidis*. We also observed additional putative SigI-associated promoters upstream of the main scaffoldin gene in *C. thermocellum*, *C. straminisolvens* JCM21531, *C. cellulovorans* 743B (ATCC 35296, DSM 3052), and *C. acetobutylicum* ATCC 824 [53]. Figure 3 shows a strong conservation of the aligned promoter sequences, and supports the hypothesis of a possible regulatory role of an extended 5′-UTR in the regulation of post-transcriptional events, which might indicate a translation step of scaffoldin expression.

### 3.5. Sequence Conservation in Cohesins and Dockerins Suggest Cross-Species Recognition

In order to compare the sequence conservation of key cellulosomal components among the mesophilic cellulolytic clostridia, namely the cohesins and dockerins, we searched bioinformatically for cohesins of the major scaffoldins from newly sequenced genomes by BLAST, using known modules as query sequences. Overall, most bacteria harbor more than ~70 dockerin-containing proteins, and less than a dozen cohesin modules, organized in a handful of scaffoldins, with the exception of *C. sufflavum* having 15 cohesins, with *C. saccharoperbutylacetonicum* having 8 dockerin-bearing enzymes, and *C. bornimense* only 5 dockerins identified in its genome (Table 1).

**Figure 4:**
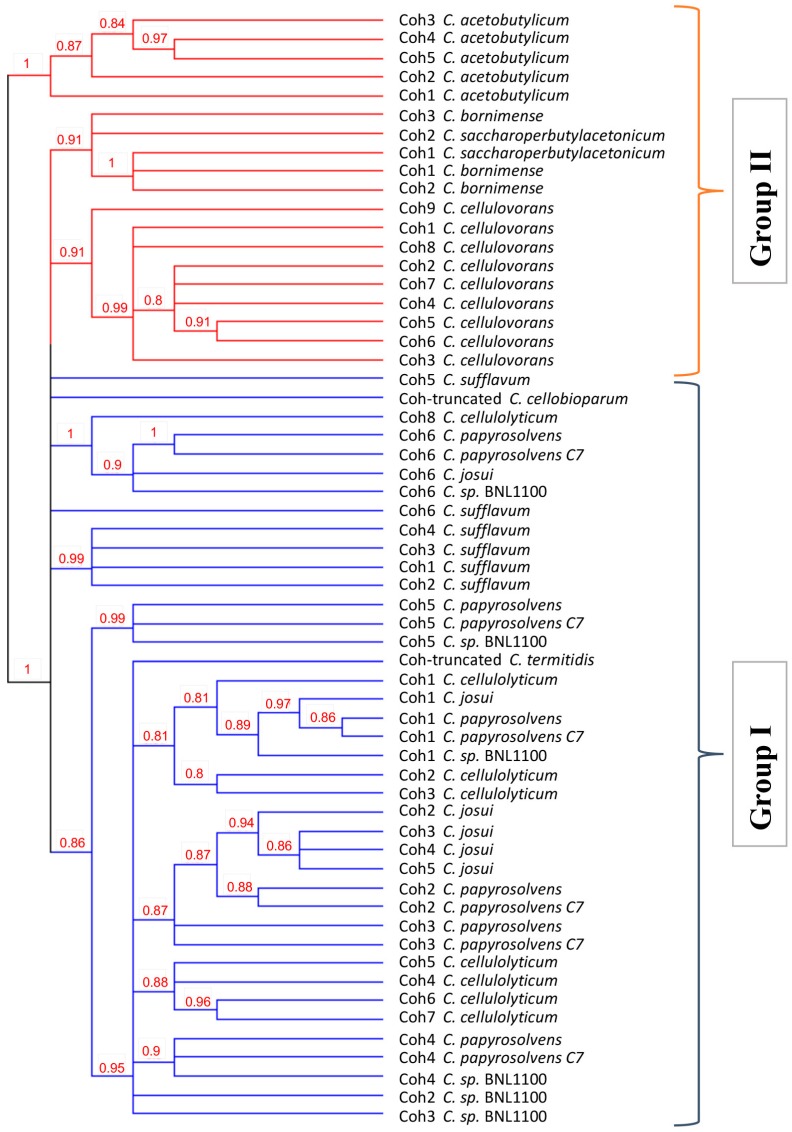
Phylogenetic relation of cohesin modules from the major scaffoldins of mesophilic cellulolytic clostridia. Protein sequences of major scaffoldin cohesins were aligned and analyzed by PhyML. Bootstrap values are denoted, and branches below 80% bootstrapping were collapsed. Two major branches of the dendogram (red and blue) separate between *C. acetobutylicum*, *C. cellulovorans*, *C. bornimense*, and *C. saccharoperbutylacetonicum* from the other mesophiles.

Analysis of the phylogenetic relationship among the 59 cohesins from the major scaffoldins of all examined species supports the distinction of two major evolutionary groups of species (red and blue branches in Figure 4). This may suggest a common ancestor for all these species, which further evolved into two distinct routes, distinguishing between the scaffoldin cohesins of *C. acetobutylicum*, *C. cellulovorans*, *C. bornimense*, and *C. saccharoperbutylacetonicum* (Group I in Figure 4) from the other mesophiles (Group II in Figure 4), and with the *C. acetobutylicum* cohesins representing the most remote group of outliers. This is in accordance with previous 16S rDNA analysis showing a distinction between *C. cellulovorans* and related sequences [55]. The dendrogram indicated that *C. papyrosolvens* cohesins are similar to those of *C. cellulolyticum* and *C. josui* (suggesting cross-species recognition), and are distinct from *C. acetobutylicum* and *C. cellulovorans* (the later are separated on different branches of the tree).

**Figure 5:**
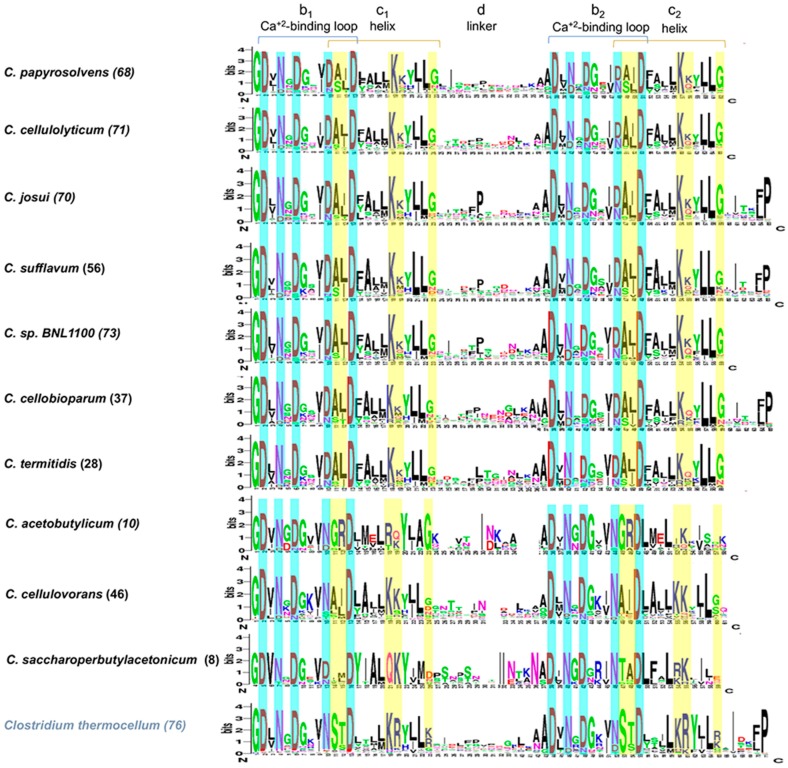
Conserved sequence features of dockerin modules in cellulolytic species. Aligned sequences of the dockerin module within each species were visualized by WebLogo. Similar profiles of dockerins were observed among the species, in particularly, the conservation of putative cohesin–dockerin binding positions at the Ca-binding loop (in yellow). Number of aligned sequences species is marked in brackets. Dockerin segments (b–d at top) are labelled according to Pagès et al. [39]. *C. bornimense* was omitted, because it contains only five sequences.

We next compared the sequence conservation of dockerin modules. The dockerin is typically a protein of ~70 amino acids long, that resides within carbohydrate-degrading enzymes, usually at the N terminus, and serves to anchor the enzyme into the cellulosome by direct interaction with cohesin modules on the scaffoldin (Figure 5). In general, the dockerin modules of the different species share high sequence similarity, and the dockerin modules of *C. cellulolyticum* and *C. papyrosolvens* show greater than 90% sequence similarity. In Figure 5, we observed that the dockerin organization is maintained among all species examined. This includes the two typically conserved repeats of calcium-binding loops followed by an “F helix”, that are connected by a variable linker region [56]. A conserved N-terminal Gly residue and the canonical pattern of Asp/Asn are kept within the cellulosomal clostridial mesophiles at the calcium-coordinating positions 1, 3, 5, 9, and 12 (Figure 5).

The nature of the “specificity determinants” (i.e., residues at positions 10, 11, 17, 18, and 22 within the repeated segment) is also preserved among the mesophiles [57,58,59]. Yet, while in the complex cellulosome of the thermophile *C. thermocellum* residues, 10/11 are usually occupied by conserved Ser/Thr (Figure 5), comparison of dockerin profiles of the mesophilic cellulosome-producing bacteria indicates conservation of Ala/Leu(Ile) in these positions instead, suggesting general, similar dockerin-binding specificities (Figure 5). Interestingly, *C. cellulovorans* shows a similar pair of residues at the 10/11 position, whereas *C. acetobutylicum* has unique residues in that position, as does *C. saccharoperbutylacetonicum*.

### 3.6. Sporadic Spatial Organization of the Cellulosomal Genes along the Bacterial Chromosome 

**Figure 6:**
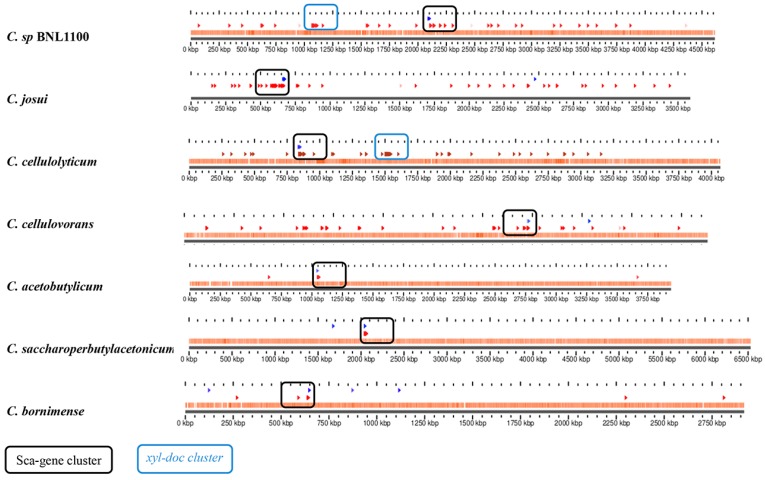
Arrangement of cohesins and dockerins along the bacterial chromosomes of cellulosome-producing mesophiles. Cohesins (blue triangles) and dockerin modules (red triangles) were searched by BLAST and located on the bacterial chromosome. Known clusters of dockerins (the *xyl-doc* cluster) and the *sca* gene cluster are marked in blue and black rectangles, respectively, whereas most other dockerin-containing genes were distributed along the chromosome.

The physical organization of the cohesin- and dockerin-containing proteins was evaluated using BLAST sequence search against each genome (Figure 6). Such an analysis was applied only on complete genome sequences or those bearing two large assembly contigs (thus excluding *C. papyrosolvens*, *C. sufflavum*, *C. cellobioparum*, and *C. termitidis* from this analysis). Most dockerin-containing genes were sporadically distributed along the chromosome in species with a high (>10) copy number of dockerins (*Clostridium* sp. BNL1100, *C. josui*, *C. cellulolyticum*, and *C. cellulovorans*), except for two gene clusters. One cluster, which appears in all species, is the *sca* gene cluster, which contains cohesins coded in the Cip scaffoldin and in the *orfX* gene, together with dockerin-containing enzymes of that operon (Figure 6). An additional cluster is the “*xyl–doc*” cluster, encoding 14 dockerin-containing hemicellulases, which was originally reported in *C. cellulolyticum* (Ccel_1229-1242) [60]. BLAST searches using this cluster showed that it is also conserved in *Clostridium* sp. BNL1100. The sporadic spatial organization of the numerous dockerin-containing genes in the genome suggests the importance of the cellulosomal paradigm in those bacterial species. However, such a conclusion could not statistically be validated for species with only a few dockerins (*C. acetobutylicum*, *C. saccharoperbutylacetonicum*, and *C. bornimense*).

### 3.7. Profiling the Carbohydrate-Active Enzymes in the Cellulosome-Producing Mesophiles

**Figure 7:**
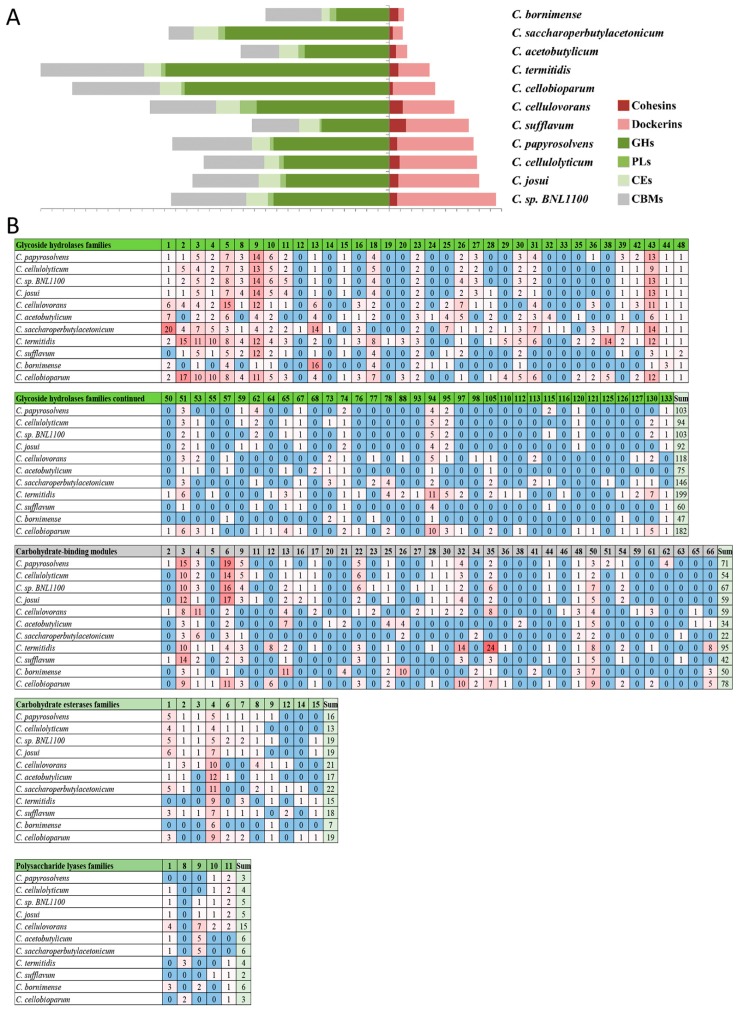
Frequency of CAZY modules identified in mesophiles. (**A**) Number of Carbohydrate-Active enZYmes (CAZyme modules) is denoted for each genome of the mesophilic clostridia. Precise numbers are available in Table 1. (**B**) A detailed count of CAZYmes and their assignment to the different family types. Glycoside hydrolases (GHs), polysaccharide lyases (PLs), carbohydrate esterases (CEs), carbohydrate-binding modules (CBM).

The identification of cellulosome-related carbohydrate active enzymes (CAZymes) is key for understanding the complex functions of carbohydrate degradation in cellulolytic bacteria. We profiled the elaborate reservoir of dockerin-containing cellulases using the comprehensive CAZy classification system [30]. This enabled the identification of numerous glycoside hydrolases (GHs), carbohydrate esterases (CEs), polysaccharide lyases (PLs), and in proteins bearing carbohydrate-binding modules (CBMs) (Figure 7). In cases in which the proteins bear a dockerin module, the latter mediates the incorporation of the cellulase into the cellulosomal scaffoldin via cohesin–dockerin interaction.

Closer analysis reveals that glycoside hydrolases (GH) contribute the major fraction to the total number of CAZymes (Figure 7A and Table 1). Notably, *C. cellulovorans* has an exceptionally high number of 15 polysaccharide lyases (PLs). Differences are also observed in the number of CBMs, ranging from 22 copies in *C. saccharoperbutylacetonicum* to 95 in *C. termitidis*, and the variation is even more pronounced regarding the cellulose-binding family 3 alone. Among the genomes analyzed, the varying number of non-catalytic modules (cohesins, CBMs) did not correlate with the number of catalytic modules (CAZymes, either with or without dockerins) (Figure 7A and Table 1). This may suggest that the complexity of a cellulosome is not a trivial statistical function of the number of modules, and that additional parameters may be involved, such as gene organization, types of binding modules or gene regulation.

The vast majority (91%) of the dockerin-containing proteins are secreted enzymes, wherein the proteins possess a signal peptide sequence (some bacteria have a unique signal peptides sequences which are often not identified by the SignalP server). A wide variety of carbohydrate-degrading modules, i.e., GHs, CEs, and PLs, can be identified in the dockerin-encoding genes, suggesting diversity in enzymatic activity. Of note are the genomes of *C. termitidis*, *C. cellobioparum* and *C. saccharoperbutylacetonicum*, which bear more than 140 GH enzymes. The catalytic modules are collectively associated with dozens of different non-catalytic CBMs, which were identified in each mesophile, and notably expanded in *C. termitidis* (Figure 7B and Table 1). In *C. papyrosolvens*, the most abundant GH families are GH5, GH9, and GH43, which constitute over 50% of the enzymatic domains identified.

While comparing the CAZomes of two very closely related cellulosome-producing mesophilic bacteria—*C. cellulolyticum* and *C. papyrolsovens* (which exhibit 98.9% similarity in their 16S rRNA sequences)—several differences could be noted. Whole-genome analysis of *C. papyrosolvens* revealed 98 GH domains and 66 CBMs, representing a notable increase, compared to the 91 and 54 domains observed in *C. cellulolyticum.* Included in the *C. papyrosolvens* GH families are GH25 and GH36, of which there are no representatives in the *C. cellulolyticum* genome (Figure 7B). Conversely, GH65 and GH73 domains are each found in single copies in *C. cellulolyticum*, but are absent in *C. papyrosolvens*. The differences in numbers may be attributed to the size of the genomes, which are 4.92 Mb for *C. papyrosolvens*, and 4.07 for *C. cellulolyticum*. Yet, these data indicate pointed diversity of CAZymes and related domains beyond the cellulosome-associated components, and suggest that, like other cellulolytic bacteria, the various individual mesophilic clostridial species have evolved several specific strategies for carbohydrate degradation, some similar to, but others distinct from those of their intimate relatives.

## 4. Discussion

In early work, selected anaerobic mesophilic bacteria were found to exhibit distinctive characteristics consistent with the production of cellulosomes [41,61]. The list of such bacteria was later extended in additional studies by the sequencing of scaffoldin genes in other mesophilic cellulolytic clostridia [37,39]. With the advent and progression of the era of genome sequencing, additional cellulosome-producing, mesophilic clostridia were discovered. Surprisingly, the different species display great similarity in their cellulosomal components, which includes the nature of their enzyme-integrating scaffoldin subunit, the types (and usually number) of dockerin-bearing enzymes, and the amino acid residues that occupy positions in the dockerin consistent with recognition of the cohesin counterpart. Moreover, several very basic cellulosome genes are contained in a telltale gene cluster on the chromosome in all of the mesophilic clostridial species, which includes genes coding for the major scaffoldin subunit, the mysterious single-cohesin-containing OrfX, the major family 48 cellulase, and other cellulases from families 5 and 9.

Our work herein links the need of a given cellulolytic bacterium to express various fibrolytic activities and the genome-wide coding of key cellulosomal components in different mesophilic cellulosome-producing bacteria. On the one hand, the current work further demonstrated the relatedness among the cellulosome-producing mesophiles, each of which possesses a simple cellulosomal architecture compared to the complex multi-scaffoldin cellulosomes of other clostridia and ruminococci. The mesophilic clostridia share common features which distinguish their cellulosomes from those of other species, such as, the similar organization of the *sca* gene cluster which was observed for *C. cellulolyticum*, *Clostridium* sp. BNL1100, *C. papyrosolvens* and *C. josui*, along with conserved functional and sequence profiles of their cohesin and dockerin modules. This similarity suggests that the *sca* gene cluster, with its collection of cellulosomal component genes, was horizontally transferred among these mesophiles from a common ancestor [26]. On the other hand, we noted differences in the type and proportions of key CAZyme components among the mesophiles. These may reflect a specialized repertoire of carbohydrate-degrading strategies, which have evolved in each bacterium, tailored for its diverse habitat, lifestyle, its physical conditions or interaction with other organisms.

The dockerin profile of the mesophilic cellulosome-producing bacteria includes the definitive repeated calcium-binding loop and adjacent helix segment, but differs in their conserved putative recognition residues from those of the complex cellulosome-producing bacteria, e.g., *C. thermocellum*, *B. cellulosolvens*, *C. clariflavum*, and *R. flavefaciens.* This may suggest collective species-specific preferences, to eliminate cross-species binding with the cohesin-bearing scaffoldins of the complex cellulosome-producing bacteria, as was observed in [62]. In contrast, most, but not all, of the cohesin–dockerin interactions of the mesophilic clostridia appear to share the same general recognition residues, which may indicate general cross-species interaction of their scaffoldins and enzyme subunits in nature, and would imply their coexistence in the same ecological niche. In any case, evolutionary forces have not proved fit to change them during speciation processes [5,63]. Unlike the majority of the mesophilic clostridia, however, distinct alternative recognition residues are evident in *C. saccharoperbutylacetonicum* and *C. bornimense.* It is also currently enigmatic why these two species have two CBM3s in their respective scaffoldin with a reduced number of cohesins and similarly reduced number of dockerin-bearing enzymes. It seems that their abridged cellulosomes would assume a supportive role to the much larger collection of free enzymes in these species. Nevertheless, the presence of typical cellulosome-based cellulases, i.e., GH48, GH9, and GH5 enzymes, may indicate their significance for the parent bacterium in the degradation of recalcitrant forms of cellulosic biomass.

Further studies are needed to elucidate the interactions of the cellulosomal components of the newly described species, such as *C. sufflavum*, *C. termitidis*, *C. saccharoperbutylacetonicum* and *C. bornimense*. This is also true for a full understanding of the role of the “inactive” cellulosome complex of *C. acetobutylicum*, which has little or no detectable cellulolytic activities, but maintains a conserved scaffoldin, dockerins, and CAZymes (including the dominant GH48 enzyme and other long-established types of cellulases [9]). Genomes of a second *C. papyrosolvens* and several other strains of *C. acetobutylicum* have also been sequenced, but were omitted from this study. Likewise, additional related cellulosome-producing mesophilic clostridia, such as *Clostridium puniceum*, *Herbinix luporum*, *Clostridium hungatei*, *Clostridium roseum*, etc., have not been included herein. Moreover, the contribution of additional, recently sequenced mesophilic, but complex, multi-scaffoldin cellulosome-producing bacteria, such as *Clostridium alkalicellulosi* and *Bacteroides* (*Pseudobacteroides*) *cellulosolvens* [19], will also shed light on the cellulosomal models of the mesophilic bacteria.

Hydrolysis of cellulosic substrates is a major biotechnological challenge. Reconstitution of the biological principle of native cellulosomes and their application as components for chimeric designer cellulosomes [64,65,66,67,68] may provide a basis for improved cellulolytic activity. The cellulosome complexes of the mesophilic clostridia contain a wealth of polypeptide modules that can be utilized for numerous applications. Cohesin and dockerin modules can also be fused to various non-cellulolytic biologically active macromolecules for use in a large range of affinity-based systems. The developing nanotechnologies will require a diversity of such “Lego”-like molecular adaptors or connecting modules. The components discovered and analyzed in each cellulsome-producing bacterium now joins the growing library of divergent cohesins, dockerins, and other cellulosome-related modules, and may contribute to future application as “spare parts” for fabrication of defined nanoassemsblies.

## Figures and Tables

**Table 1:** Cellulosomal and CAZy metrics of the *Clostridia* mesophiles analyzed in this study.

Species	Genome Accession	Genome Sequencing Level	No. of Contigs	Scaffoldins	Cohesins	Dockerins	GHs	PLs	CEs	CBMs	Total CAZYmes	Source	References
*Clostridium* sp. BNL1100	CP003259.1	Complete	1	2	7	88	103	5	19	67	127	Corn stover	[42]
*C. josui* JCM17888	JAGE00000000.1	Draft	2	3	8	72	92	5	19	59	116	Compost	[37]
*C. cellulolyticum H10*	CP001348.1	Complete	1	2	9	69	94	4	13	54	111	Compost	[39]
*C. papyrosolvens DSM 2782*	ACXX00000000.2	Draft	31	2	7	68	103	3	16	71	122	Paper mill	[38]
*C. sufflavum* DSM 19573	PRJNA262320	Draft	57	5 (6)	15	56	60	2	18	42	80	Methanogenic reactor	[47]
*C. cellulovorans* 743B	CP002160.1	Complete	1	5	12	46	118	15	21	59	154	Wood fermenter	[40]
*C. cellobioparum* DSM 1351	JHYD01000000.1	Draft	80	3	3	38	182	3	19	78	204	Rumen of cattle	[41]
*C. termitidis* CT1112	AORV00000000.1	Draft	78	7	8	28	199	4	15	95	218	Gut of termite	[46]
*C. acetobutylicum* DSM 1731	CP002660.1	Complete	1	2	6	10	75	6	17	34	98	Soil	[36]
*C. saccharoperbutylacetonicum* N1-4 (HMT)	CP004121.1	Complete	1	2	3	9	146	6	22	22	174	Soil	[45]
*C. bornimense* (=*Clostridium* sp. M2/40)	HG917868.1, HG917869.1	Draft	2	2	8	5	47	6	7	50	60	Biogas reactor	[43]

## Supplementary Materials

The following are available online at www.mdpi.com/2076-2607/5/4/74/s1.

## Abbreviations

CBM: carbohydrate binding module; CE: carbohydrate esterase; GH: glycoside hydrolase; PL: polysaccharide lyase; ORF, open-reading frame.

## References

[1] Albersheim P., Darvill A., Roberts K., Sederoff R., Staehelin A. (2010). Plant Cell Walls.

[2] Artzi L., Bayer E.A., Moraïs S. (2017). Cellulosomes: Bacterial nanomachines for dismantling plant polysaccharides. Nat. Rev. Microbiol..

[3] Schwarz W.H. (2001). The cellulosome and cellulose degradation by anaerobic bacteria. Appl. Microbiol. Biotechnol..

[4] Himmel M.E., Xu Q., Luo Y., Ding S.-Y., Lamed R., Bayer E.A. (2010). Microbial enzyme systems for biomass conversion: Emerging paradigms. Biofuels.

[5] Bayer E.A., Shoham Y., Lamed R., Rosenberg E. (2013). Lignocellulose-decomposing bacteria and their enzyme systems. The Prokaryotes.

[6] Doi R.H. (2008). Cellulases of mesophilic microorganisms: Cellulosome and noncellulosome producers. Ann. N. Y. Acad. Sci..

[7] Bayer E.A., Belaich J.-P., Shoham Y., Lamed R. (2004). The cellulosomes: Multi-enzyme machines for degradation of plant cell wall polysaccharides. Annu. Rev. Microbiol..

[8] Belaich J.-P., Tardif C., Belaich A., Gaudin C. (1997). The cellulolytic system of *Clostridium cellulolyticum*. J. Biotechnol..

[9] Sabathe F., Belaich A., Soucaille P. (2002). Characterization of the cellulolytic complex (cellulosome) of *Clostridium acetobutylicum*. FEMS Microbiol. Lett..

[10] Tamaru Y., Karita S., Ibrahim A., Chan H., Doi R.H. (2000). A large gene cluster for the *Clostridium cellulovorans* cellulosome. J. Bacteriol..

[11] Sakka M., Goto M., Fujino T., Fujino E., Karita S., Kimura T., Sakka K. (2010). Analysis of a *Clostridium josui* cellulase gene cluster containing the *man5a* gene and characterization of recombinant Man5A. Biosci. Biotechnol. Biochem..

[12] Poole D.M., Morag E., Lamed R., Bayer E.A., Hazlewood G.P., Gilbert H.J. (1992). Identification of the cellulose binding domain of the cellulosome subunit S1 from *Clostridium thermocellum*. FEMS Microbiol. Lett..

[13] Mosbah A., Belaich A., Bornet O., Belaich J.P., Henrissat B., Darbon H. (2000). Solution structure of the module X2_1 of unknown function of the cellulosomal scaffolding protein CipC of *Clostridium cellulolyticum*. J. Mol. Biol..

[14] Vazana Y., Barak Y., Unger T., Peleg Y., Ben Yehezkel T., Mazor Y., Shapiro E., Lamed R., Bayer E.A. (2013). A synthetic biology approach for evaluating the functional contribution of designer cellulosome components to deconstruction of cellulosic substrates. Biotechnol. Biofuels.

[15] Bayer E.A., Smith S.P., Noach I., Alber O., Adams J.J., Lamed R., Shimon L.J.W., Frolow F., Sakka K., Karita S., Kimura T., Sakka M., Matsui H., Miyake H., Tanaka A. (2009). Can we crystallize a cellulosome?. Biotechnology of Lignocellulose Degradation and Biomass Utilization.

[16] Fujino T., Béguin P., Aubert J.-P. (1993). Organization of a *Clostridium thermocellum* gene cluster encoding the cellulosomal scaffolding protein cipa and a protein possibly involved in attachment of the cellulosome to the cell surface. J. Bacteriol..

[17] Lamed R., Setter E., Bayer E.A. (1983). Characterization of a cellulose-binding, cellulase-containing complex in *Clostridium thermocellum*. J. Bacteriol..

[18] Xu Q., Bayer E.A., Goldman M., Kenig R., Shoham Y., Lamed R. (2004). Architecture of the *Bacteroides cellulosolvens* cellulosome: Description of a cell-surface anchoring scaffoldin and a family-48 cellulase. J. Bacteriol..

[19] Zhivin O., Dassa B., Moraïs S., Uttukar S.M., Brown S.D., Henrissat B., Lamed R., Bayer E.A. (2017). Unique organization and unprecedented diversity of the *Bacteroides (Pseudobacteroides) cellulosolvens* cellulosome system. Biotechnol. Biofuels.

[20] Dassa B., Borovok I., Lamed R., Henrissat B., Coutinho P., Hemme C.L., Huang Y., Zhou Z., Bayer E.A. (2012). Genome-wide analysis of *Acetivibrio cellulolyticus* provides a blueprint of an elaborate cellulosome system. BMC Genom..

[21] Hamberg Y., Ruimy-Israeli V., Dassa B., Barak Y., Lamed R., Cameron K., Fontes C.M., Bayer E.A., Fried D.B. (2014). Elaborate cellulosome architecture of *Acetivibrio cellulolyticus* revealed by selective screening of cohesin-dockerin interactions. PeerJ.

[22] Israeli-Ruimy V., Bule P., Jindou S., Dassa B., Barak Y., Slutzki M., Hamberg Y., Cardoso V., Alves V.D., Najmudin S. (2017). Complexity of the *Ruminococcus flavefaciens* FD-1 cellulosome reflects an expansion of family-related protein-protein interactions. Sci. Rep..

[23] Rincon M.T., Cepeljnik T., Martin J.C., Barak Y., Lamed R., Bayer E.A., Flint H.J. (2007). A novel cell surface-anchored cellulose-binding protein encoded by the *sca* gene cluster of *Ruminococcus flavefaciens*. J. Bacteriol..

[24] Artzi L., Dassa B., Borovok I., Shamshoum M., Lamed R., Bayer E.A. (2014). Cellulosomics of the cellulolytic thermophile *Clostridium clariflavum*. Biotechnol. Biofuels.

[25] Artzi L., Morag E., Barak Y., Lamed R., Bayer E.A. (2015). *Clostridium clariflavum:* Key cellulosome players are revealed by proteomic analysis. mBio.

[26] Tamaru Y., Miyake H., Kuroda K., Nakanishi A., Matsushima C., Doi R.H., Ueda M. (2011). Comparison of the mesophilic cellulosome-producing *Clostridium cellulovorans* genome with other cellulosome-related clostridial genomes. Microb. Biotechnol..

[27] Udaondo Z., Duque E., Ramos J.L. (2017). The pangenome of the genus *Clostridium*. Environ. Microbiol..

[28] Xu C., Huang R., Teng L., Jing X., Hu J., Cui G., Wang Y., Cui Q., Xu J. (2015). Cellulosome stoichiometry in *Clostridium cellulolyticum* is regulated by selective RNA processing and stabilization. Nat. Commun..

[29] Altschul S.F., Madden T.L., Schaffer A.A., Zhang J., Zhang Z., Miller W., Lipman D.J. (1997). Gapped blast and psi-blast: A new generation of protein database search programs. Nucleic Acids Res..

[30] Lombard V., Golaconda Ramulu H., Drula E., Coutinho P.M., Henrissat B. (2014). The carbohydrate-active enzymes database (CAZy). Nucleic Acids Res..

[31] Marchler-Bauer A., Bo Y., Han L., He J., Lanczycki C.J., Lu S., Chitsaz F., Derbyshire M.K., Geer R.C., Gonzales N.R. (2017). Cdd/sparcle: Functional classification of proteins via subfamily domain architectures. Nucleic Acids Res..

[32] Sievers F., Wilm A., Dineen D., Gibson T.J., Karplus K., Li W., Lopez R., McWilliam H., Remmert M., Soding J. (2011). Fast, scalable generation of high-quality protein multiple sequence alignments using Clustal Omega. Mol. Syst. Biol..

[33] Crooks G.E., Hon G., Chandonia J.M., Brenner S.E. (2004). Weblogo: A sequence logo generator. Genome Res..

[34] Aziz R.K., Bartels D., Best A.A., DeJongh M., Disz T., Edwards R.A., Formsma K., Gerdes S., Glass E.M., Kubal M. (2008). The rast server: Rapid annotations using subsystems technology. BMC Genom..

[35] Lefort V., Longueville J.E., Gascuel O. (2017). SMS: Smart model selection in PhyML. Mol. Biol. Evol..

[36] Nolling J., Breton G., Omelchenko M.V., Makarova K.S., Zeng Q., Gibson R., Lee H.M., Dubois J., Qiu D., Hitti J. (2001). Genome sequence and comparative analysis of the solvent-producing bacterium *Clostridium acetobutylicum*. J. Bacteriol..

[37] Kakiuchi M., Isui A., Suzuki K., Fujino T., Fujino E., Kimura T., Karita S., Sakka K., Ohmiya K. (1998). Cloning and DNA sequencing of the genes encoding *Clostridium josui* scaffolding protein CipA and cellulase CelD and identification of their gene products as major components of the cellulosome. J. Bacteriol..

[38] Pohlschröder M., Leschine S.B., Canale-Parola E., Shimada K., Hoshino S., Ohmiya K., Sakka K., Kobayashi Y., Karita S. (1993). Regulation of the multicomplex cellulase-xylanase system of *Clostridium papyrosolvens*. Genetics, Biochemistry and Ecology of Lignocellulose Degradation.

[39] Pagès S., Belaich A., Belaich J.-P., Morag E., Lamed R., Shoham Y., Bayer E.A. (1997). Species-specificity of the cohesin-dockerin interaction between *Clostridium thermocellum* and *Clostridium cellulolyticum:* Prediction of specificity determinants of the dockerin domain. Proteins.

[40] Sleat R., Mah R.A., Robinson R. (1984). Isolation and characterization of an anaerobic, cellulolytic bacterium, *Clostridium cellulovorans*, sp. nov.. Appl. Environ. Microbiol..

[41] Lamed R., Naimark J., Morgenstern E., Bayer E.A. (1987). Specialized cell surface structures in cellulolytic bacteria. J. Bacteriol..

[42] Li L.L., Taghavi S., Izquierdo J.A., van der Lelie D. (2012). Complete genome sequence of *Clostridium* sp. Strain BNL1100, a cellulolytic mesophile isolated from corn stover. J. Bacteriol..

[43] Hahnke S., Striesow J., Elvert M., Mollar X.P., Klocke M. (2014). *Clostridium bornimense* sp. nov., isolated from a mesophilic, two-phase, laboratory-scale biogas reactor. Int. J. Syst. Evol. Microbiol..

[44] Hahnke S., Wibberg D., Tomazetto G., Puhler A., Klocke M., Schluter A. (2014). Whole genome sequence of *Clostridium bornimense* strain M2/40 isolated from a lab-scale mesophilic two-phase biogas reactor digesting maize silage and wheat straw. J. Biotechnol..

[45] Poehlein A., Krabben P., Durre P., Daniel R. (2014). Complete genome sequence of the solvent producer *Clostridium saccharoperbutylacetonicum* strain DSM 14923. Genome Announc..

[46] Lal S., Ramachandran U., Zhang X., Munir R., Sparling R., Levin D.B. (2013). Draft genome sequence of the cellulolytic, mesophilic, anaerobic bacterium *Clostridium termitidis* strain CT1112 (DSM 5398). Genome Announc..

[47] Nishiyama T., Ueki A., Kaku N., Ueki K. (2009). *Clostridium sufflavum* sp. nov., isolated from a methanogenic reactor treating cattle waste. Int. J. Syst. Evol. Microbiol..

[48] Zverlov V.V., Kellermann J., Schwarz W.H. (2005). Functional subgenomics of *Clostridium thermocellum* cellulosomal genes: Identification of the major catalytic components in the extracellular complex and detection of three new enzymes. Proteomics.

[49] Morag E., Halevy I., Bayer E.A., Lamed R. (1991). Isolation and properties of a major cellobiohydrolase from the cellulosome of *Clostridium thermocellum*. J. Bacteriol..

[50] Ravachol J., Borne R., Tardif C., de Philip P., Fierobe H.P. (2014). Characterization of all family-9 glycoside hydrolases synthesized by the cellulosome-producing bacterium *Clostridium cellulolyticum*. J. Biol. Chem..

[51] Tomazetto G., Hahnke S., Koeck D.E., Wibberg D., Maus I., Pühler A., Klocke M., Schluter A. (2016). Complete genome analysis of *Clostridium bornimense* strain M2/40(T): A new acidogenic *Clostridium* species isolated from a mesophilic two-phase laboratory-scale biogas reactor. J. Biotechnol..

[52] Abdou L., Boileau C., de Philip P., Pages S., Fierobe H.P., Tardif C. (2008). Transcriptional regulation of the *Clostridium cellulolyticum cip-cel* operon: A complex mechanism involving a catabolite-responsive element. J. Bacteriol..

[53] Asai K., Ootsuji T., Obata K., Matsumoto T., Fujita Y., Sadaie Y. (2007). Regulatory role of RsgI in *sigI* expression in *Bacillus subtilis*. Microbiology.

[54] Ortiz de Ora L., Munoz-Gutierrez I., Bayer E.A., Shoham Y., Lamed R., Borovok I. (2017). Revisiting the regulation of the primary scaffoldin gene in *Clostridium thermocellum*. Appl. Environ. Microbiol..

[55] Rainey F.A., Stackebrandt E. (1993). 16S rDNA analysis reveals phylogenetic diversity among the polysaccharolytic clostridia. FEMS Microbiol. Lett..

[56] Gal L., Pagès S., Gaudin C., Belaich A., Reverbel-Leroy C., Tardif C., Belaich J.-P. (1997). Characterization of the cellulolytic complex (cellulosome) produced by *Clostridium cellulolyticum*. Appl. Environ. Microbiol..

[57] Mechaly A., Yaron S., Lamed R., Fierobe H.P., Belaich A., Belaich J.P., Shoham Y., Bayer E.A. (2000). Cohesin-dockerin recognition in cellulosome assembly: Experiment versus hypothesis. Proteins.

[58] Schaeffer F., Matuschek M., Guglielmi G., Miras I., Alzari P.M., Béguin P. (2002). Duplicated dockerin subdomains of *Clostridium thermocellum* endoglucanase CelD bind to a cohesin domain of the scaffolding protein CipA with distinct thermodynamic parameters and a negative cooperativity. Biochemistry.

[59] Pinheiro B.A., Proctor M.R., Martinez-Fleites C., Prates J.A., Money V.A., Davies G.J., Bayer E.A., Fontesm C.M., Fierobe H.P., Gilbert H.J. (2008). The *Clostridium cellulolyticum* dockerin displays a dual binding mode for its cohesin partner. J. Biol. Chem..

[60] Xu C., Huang R., Teng L., Wang D., Hemme C.L., Borovok I., He Q., Lamed R., Bayer E.A., Zhou J. (2013). Structure and regulation of the cellulose degradome in *Clostridium cellulolyticum*. Biotechnol. Biofuels.

[61] Lamed R., Bayer E.A., Aubert J.-P., Beguin P., Millet J. (1988). The cellulosome concept: Exocellular/extracellular enzyme reactor centers for efficient binding and cellulolysis. Biochemistry and Genetics of Cellulose Degradation.

[62] Haimovitz R., Barak Y., Morag E., Voronov-Goldman M., Lamed R., Bayer E.A. (2008). Cohesin-dockerin microarray: Diverse specificities between two complementary families of interacting protein modules. Proteomics.

[63] Bayer E.A., Shoham Y., Lamed R., Doyle R.J. (2000). The cellulosome—An exocellular organelle for degrading plant cell wall polysaccharides. Glycomicrobiology.

[64] Bayer E.A., Morag E., Lamed R. (1994). The cellulosome—A treasure-trove for biotechnology. Trends Biotechnol..

[65] Davidi L., Moraïs S., Artzi L., Knop D., Hadar Y., Arfi Y., Bayer E.A. (2016). Toward combined delignification and saccharification of wheat straw by a laccase-containing designer cellulosome. Proc. Natl. Acad. Sci. USA.

[66] Fierobe H.-P., Mingardon F., Mechaly A., Belaich A., Rincon M.T., Lamed R., Tardif C., Belaich J.-P., Bayer E.A. (2005). Action of designer cellulosomes on homogeneous versus complex substrates: Controlled incorporation of three distinct enzymes into a defined tri-functional scaffoldin. J. Biol. Chem..

[67] Moraïs S., Morag E., Barak Y., Goldman D., Hadar Y., Lamed R., Shoham Y., Wilson D.B., Bayer E.A. (2012). Deconstruction of lignocellulose into soluble sugars by native and designer cellulosomes. mBio.

[68] Stern J., Moraïs S., Lamed R., Bayer E.A. (2016). Adaptor scaffoldins: An original strategy for extended designer cellulosomes, inspired from nature. mBio.

